# Wildtype epidermal growth factor receptor (*Egfr*) is not required for daily locomotor or masking behavior in mice

**DOI:** 10.1186/1740-3391-4-15

**Published:** 2006-11-16

**Authors:** Reade B Roberts, Carol L Thompson, Daekee Lee, Richard W Mankinen, Aziz Sancar, David W Threadgill

**Affiliations:** 1 Department of Genetics, CB 7264, University of North Carolina at Chapel Hill, Chapel Hill, NC 27599, USA; 2 Department of Biochemistry, CB 7260, University of North Carolina at Chapel Hill, Chapel Hill, NC 27599, USA

## Abstract

**Background:**

Recent studies have implicated the epidermal growth factor receptor (EGFR) within the subparaventricular zone as being a major mediator of locomotor and masking behaviors in mice. The results were based on small cohorts of mice homozygous for the hypomorphic *Egfr*^*wa*2 ^allele on a mixed, genetically uncontrolled background, and on intraventricular infusion of exogenous EGFR ligands. Subsequenlty, a larger study using the same genetically mixed background failed to replicate the original findings. Since both previous approaches were susceptible to experimental artifacts related to an uncontrolled genetic background, we analyzed the locomotor behaviors in *Egfr*^*wa*2 ^mutant mice on genetically defined, congenic backgrounds.

**Methods:**

Mice carrying the *Egfr*^*wa*2 ^hypomorphic allele were bred to congenicity by backcrossing greater than ten generations onto C57BL/6J and 129S1/SvImJ genetic backgrounds. Homozygous *Egfr*^*wa*2 ^mutant and wildtype littermates were evaluated for defects in locomotor and masking behaviors.

**Results:**

Mice homozygous for *Egfr*^*wa*2 ^showed normal daily locomotor activity and masking indistinguishable from wildtype littermates at two light intensities (200–300 lux and 400–500 lux).

**Conclusion:**

Our results demonstrate that reduced EGFR activity alone is insufficient to perturb locomotor and masking behaviors in mice. Our results also suggest that other uncontrolled genetic or environmental parameters confounded previous experiments linking EGFR activity to daily locomotor activity and provide a cautionary tale for genetically uncontrolled studies.

## Background

The epidermal growth factor receptor (EGFR) pathway plays key roles in the development and maintenance of many tissue and organ systems [1]. Recent reports have suggested that the EGFR pathway mediates two aspects of behavior, diurnal locomotor activity and suppression of locomotion in response to light (masking). Levels of the EGFR ligand transforming growth factor alpha (TGFA) fluctuate with a circadian rhythm within the suprachiasmatic nucleus (SCN) [2,3], which is located within the hypothalamus and is considered the primary anatomical circadian clock, and are associated with circadian time-dependent changes in gene expression [4]; similarly, EGFR ligands are expressed within cells of the retina, which modulates masking behavior [3]. Both of these structures appear to input into the subparaventricular zone (SPZ), a hypothalamic region that is required for circadian rhythms [5] and that expresses high levels of EGFR [3]. This anatomical network has been experimentally manipulated, with infusion of TGFA into the hamster hypothalamus reversibly suppressing locomotor activity [3,6]. However, exogenous administration of receptor ligands can lead to non-physiological responses, indicating what a protein can do, not necessarily its normal biological function [7].

Perhaps the most compelling evidence implicating the EGFR pathway in locomotor activity and masking was found in the behavior of mice homozygous for the *Egfr*^*wa*2 ^allele, which produces a hypomorphic receptor with reduced kinase activity [8,9]. The *Egfr*^*wa*2 ^allele is a valuable genetic reagent for dissecting biological functions of EGFR since homozygosity for *Egfr *null alleles results in lethality [1,10], while *Egfr*^*wa*2 ^homozygous animals can survive to adulthood. Both abnormally high diurnal activity and strong masking defects were reported in four of five B6EiC3H-a/A-*Egfr*^*wa*2/*wa*2 ^*Wnt3a*^*vt*/*vt *^mice tested [3]. Conflicting with these observations, a larger study using a greater number of mice from the same source (The Jackson Laboratory) and a range of illumination intensities did not detect any masking defects [11].

Previous analyses investigating alterations in locomotor and masking behaviors used mice carrying the *Egfr*^*wa*2 ^allele on a mixed genetic background. In order to resolve the discrepancies implicating a critical role for EGFR in locomotor and masking behaviors, as well as to examine potential genetic background effects on these defects, we tested *Egfr*^*wa*2 ^homozygotes on uniform congenic or hybrid backgrounds for locomotor activity and masking ability. The current results conclusively demonstrate that EGFR is not an essential mediator of locomotor or masking behaviors and suggests that other uncontrolled genetic or environmental parameters confounded previous experiments.

## Methods

B6EiC3H-a/A-*Egfr*^*wa*2/*wa*2 ^*Wnt3a*^*vt*/*vt *^mice were obtained from The Jackson Laboratory (Bar Harbor, ME). The derivation of the *Egfr*^*wa*2 ^congenic lines involved backcrossing the *Egfr*^*wa*2 ^allele for greater than ten generations to C57BL/6J and 129S1/SvImJ genetic backgrounds. The removal of the linked *Wnt3a*^*vt *^hypomorphic allele, maintained in cis with *Egfr*^*wa*2 ^in the initial starting population, was verified by PCR-based genotyping. Mice were given at least one week to acclimate to their new environment prior to testing. Mice were provided PicoLab Mouse Diet 20 (LabDiet) *ad libitum and *autoclaved water.

Mice were housed singly and habituated several days in cages equipped with running wheels for activity measurements, within a light-tight box containing a cool-white fluorescent tube providing 200 to 300 lux or 400 to 500 lux light at cage level on a 12 h:12 h light-dark cycle. Diurnal activity was calculated as the percentage of total activity occurring in the light phase of the cycle. For the masking response analysis, either one or three hour light pulses were delivered on the third day starting at Zeitgeber Time (ZT) 14 (ZT0 = light on; ZT12 = lights off). Each animal was tested for masking to two or three light pulses with at least two days between pulses. Activity during the pulse was calculated as a percentage of the activity at the same time on the previous day. Locomotor activity was recorded and analyzed with ClockLab software (Actimetrics, Evanston, IL).

## Results and discussion

Mice with normal light-mediated locomotor activity generally have one to two percent daytime activity, and masking of greater than 95%. An initial round of testing utilized commonly administered conditions, one-hour light pulses at 400–500 lux, to determine masking response. Of the six animals tested, none showed a masking defect, and only a single animal, an *Egfr*^*wa*2/*wa*2 ^*Wnt3a*^*vt*/*vt *^mouse on a mixed genetic background similar to those previously used [3], demonstrated higher than normal diurnal activity (data not shown).

**Figure 1:**
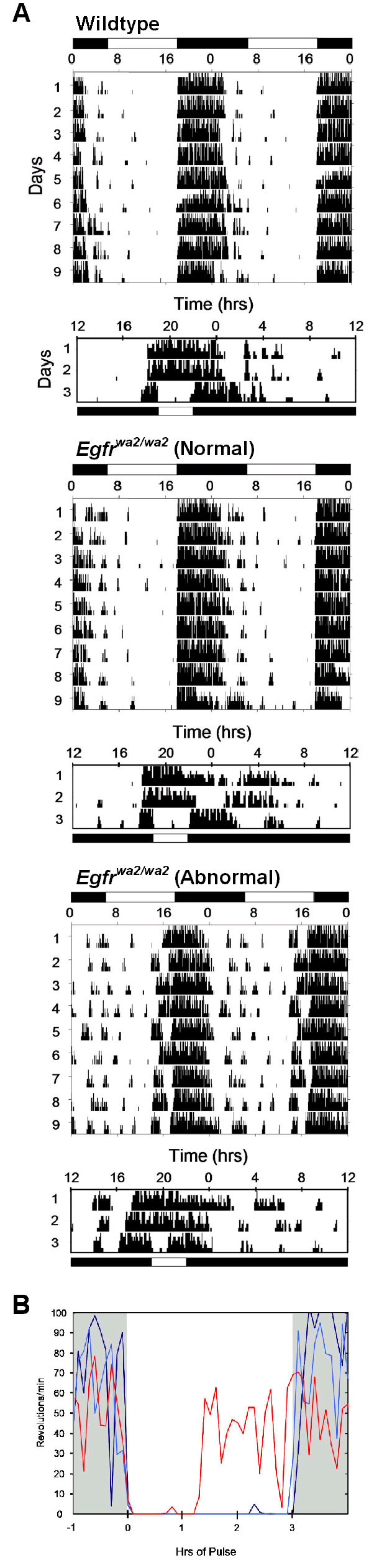
**Locomotor activity in *Egfr*^*wa*2/*wa*2 ^mice during 12 h:12 h light:dark cycles and three-hour light pulses given during the dark cycle**. Horizontal white and black bars represent light and dark exposure, respectively. Vertical axis indicates wheel-running activity. (A) The majority of *Egfr*^*wa*2/*wa*2 ^mice tested were indistinguishable from wildtype controls in behavior, though two *Egfr*^*wa*2/*wa*2 ^mice did exhibit a phase shift resulting in abnormally high daytime activity, as well as abnormally high activity during a three hour light pulse. (B) Abnormal wheel running activity during three-hour light pulses followed a characteristic one-hour of activity suppression in three *Egfr*^*wa*2/*wa*2 ^mice (red), while wildtype (dark blue) and the majority of *Egfr*^*wa*2/*wa*2 ^mice (light blue) demonstrated suppression of activity throughout the pulse. Grey areas, lights off; white area, light pulse.

A larger panel of mice was then tested using three-hour light pulses at 200–300 lux, conditions identical to those of the previous study reporting *Egfr*^*wa*2^-associated abnormalities [3]. Surprisingly, the vast majority of *Egfr*^*wa*2 ^homozygous mice exhibited both normal daytime activity and negative masking, as did all wild-type littermates and a C57BL/6J-*Wnt3a*^*vt*/*vt *^mouse (Table 1). Indeed, no statistically significant differences were found when the data were grouped by *Egfr *status (one-way ANOVA: % daytime activity, *p *= 0.32; % masking, *p *= 0.27; total activity (rev), *p *= 0.49). Two *Egfr*^*wa*2/*wa*2 ^mice, one male C57BL/6J-*Egfr*^*wa*2/*wa*2 ^and one female B6.129 F1-*Egfr*^*wa*2/*wa*2^, exhibited both abnormal daytime activity and negative masking defects similar, but not as extreme, as those previously described [3]. Unlike what happened in the previous study, the majority of the abnormally high daytime activity seen in the two affected mice was not sporadic, but rather occurred in a discrete time period anticipating the dark cycle and thus resulting in a consistent phase shift (Fig. 1A). Similar to the previous study, the negative masking in mice was delayed, with normal suppression of activity in the first hour of the three-hour light pulse (Fig. 1B). Thus, the current results on controlled genetic backgrounds show substantially less penetrance and expressivity than originally reported (18% versus 80% penetrance) [3].

**Table 1:** Activity measurements at 200–300 lux

Mouse number	Strain	Genotype	Total activity^a^	Daytime activity^b^	Masking^c^
1	C57BL/6J	+	30154	1.82	99.33
2	C57BL/6J	+	37951	0.28	99.45
3	C57BL/6J	+	44485	0.86	97.35
4	C57BL/6J	Wnt3a^vt/vt^	23509	1.43	99.72
5	B6.129 F1	+	51014	1.44	95.82
6	B6.129 F1	+	39133	0.74	96.10
7	C57BL/6J	Egfr^wa2/wa2^	27630	30.22	66.25
8	C57BL/6J	Egfr^wa2/wa2^	21821	1.30	95.82
9	C57BL/6J	Egfr^wa2/wa2^	40884	0.08	99.97
10	C57BL/6J	Egfr^wa2/wa2^	33975	1.16	97.80
11	C57BL/6J	Egfr^wa2/wa2^	31997	0.55	97.97
12	C57BL/6J	Egfr^wa2/wa2^	38165	1.95	80.49
13	C57BL/6J	Egfr^wa2/wa2^	35818	0.98	99.37
14	C57BL/6J	Egfr^wa2/wa2^	37774	0.39	99.27
15	C57BL/6J	Egfr^wa2/wa2^	21888	1.92	97.39
16	B6.129 F1	Egfr^wa2/wa2^	43930	17.55	26.63
17	B6.129 F1	Egfr^wa2/wa2^	46505	1.41	97.50

^a ^Average number of wheel revolutions per twenty-four hour period.

^b ^Percentage of total wheel revolutions during light cycle.

^c ^Percent suppression of activity during three-hour light pulse given during the dark cycle, relative to activity in the same dark cycle time period on the previous day.

The discrepancy between previous results using the B6EiC3H mixed genetic background and our current results with congenic mice is particularly surprising since most abnormal phenotypes increase in severity with inbreeding, this being particularly striking for phenotypes associated with *Egfr *[1,12]. However, use of the B6EiC3H-a/A-*Egfr*^*wa*2 ^*Wnt3a*^*vt *^stock to study effects of the *Egfr*^*wa*2 ^allele is immediately problematic since *Egfr*^*wa*2 ^homozygotes are also homozygous for the linked *Wnt3a*^*vt *^mutation, a hypomorphic allele of *Wnt3a *known for producing the vestigial tail phenotype [13]. Since *Wnt3a*-deficient mice exhibit defects in the hippocampus and central nervous system [14], the *Wnt3a*^*vt *^allele is possibly responsible for the activity defects. Additionally, the non-inbred B6EiC3H background, maintained through a cross-outcross mating scheme, harboring the *Egfr*^*wa*2 ^and *Wnt3a*^*vt *^alleles in cis segregates known and unknown mutations from the C57BL/6JEi and C3H/HeSnJ strains [15]. Consequently, defects like those previously reported for locomotor activity cannot be attributed to *Egfr *because an appropriate control does not exist for the mixed B6EiC3H background.

Wildtype inbred mice from the C57BL/6JEi and C3H/HeSnJ strains have different masking thresholds and vary in diurnal locomotor activity in a manner that is likely multigenic [16,17]. One strong candidate for such a modifier mutation is *retinal degeneration *(*Pdeb*^*rd*1^), which is carried by the C3H/HeSnJ strain but not C57BL/6EiJ, and causes progressive and selective degeneration of photoreceptor cells [18,19]; the *Pdeb*^*rd*1 ^mutation alone has a highly significant effect on masking behavior [16]. Melatonin production is also vastly different between the two strains contributing to the B6EiC3H mixed background, with C3H mice exhibiting high, rhythmic melatonin levels, and C57BL/6 mice exhibiting low to undetectable melatonin levels caused by mutation of at least one gene (N-acetyltransferase 2) related to melatonin production [20,21]. The previous *Egfr*^*wa*2 ^homozygous cohort that had a high penetrance of activity defects may have fortuitously contained a high frequency of these or other genetic modifiers given the small number of individuals tested.

Previous studies utilizing direct infusion of ligand to hyperactivate EGFR in the brain have shown that abnormally high EGFR signaling is capable of perturbing light-associated locomotor activity control [3,6]. However, such an approach can produce non-physiological responses that are artifactual or neomorphic in nature rather than being representative of normal biology [7]. Thus, the suggestion that locomotor activity is strongly dependent on normal levels of EGFR signaling is not supported, especially since extensive testing of B6EiC3H-*a/A Egfr*^*wa*2/*wa*2 ^*Wnt3a*^*vt*/*vt *^mice across a wide range of lighting conditions failed to detect any differences in masking response [11]. Using appropriately controlled genetic conditions, our results conclusively demonstrate that EGFR activity is not required to produce and is not a major mediator of abnormal activity phenotypes, and that other environmental, genetic, or stochastic effects are required to reveal abnormal phenotypes. A recent report revealed significant intrastrain and intraindividual fluctuations of EGFR ligand levels in the SCN of inbred mice [2]. Thus we cannot eliminate the possibility that homozygosity for *Egfr*^*wa*2 ^may cause individuals to be sensitized to phenotypically express abnormal locomotor activities. For example, *Egfr*^*wa*2 ^homozygotes have variable eye defects [8] that could combine with other factors to contribute to light-associated activity abnormalities. In fact one of the *Egfr*^*wa*2/*wa*2 ^individuals in our study supports the presence of intraindividual variability, exhibiting an abnormal masking response in one trial followed by a near-normal response in a subsequent trial (data not shown).

## Conclusion

The mice used to originally implicate EGFR as a major mediator of locomotor activity carried numerous other mutations and allelic variants that could contribute to the observed results. Since the animals were not properly controlled for genetic background, numerous other interpretations (such as unequal genetic background distribution in control and test mice) most likely contributed to the erroneous results. Our data using genetically uniform congenic lines indicate that EGFR is not a required mediator of the locomotor or masking behaviors, though it may modulate the activity of other pathways involved in control of locomotor activity. Further investigation is required to properly elucidate the molecular pathways and factors mediating locomotor activity.

## Competing interests

The author(s) declare that they have no competing interests.

## Authors' contributions

RBW participated in all experiments, in the analysis and discussion of the results, and in the writing of the manuscript. CLT participated in all experiments, in the analysis and discussion of the results, and in the writing of the manuscript. DL participated in all experiments and in the analysis and discussion of the results. RWM participated in all experiments and in the analysis and discussion of the results. AS participated in the conceptualization of the experiments and in the analysis and discussion of the results. DWT participated in the conceptualization of the experiments, in the analysis and discussion of the results, and in the writing of the manuscript. All authors read and approved the final manuscript.
